# Generation of novel trimeric fragments of human SP-A and SP-D after recombinant soluble expression in *E. coli*

**DOI:** 10.1016/j.imbio.2020.151953

**Published:** 2020-07

**Authors:** Alastair Watson, Grith L. Sørensen, Uffe Holmskov, Harry J. Whitwell, Jens Madsen, Howard Clark

**Affiliations:** a Department of Child Health, Division of Clinical and Experimental Sciences, Faculty of Medicine, University of Southampton, Southampton General Hospital, Southampton, United Kingdom; b Institute of Molecular Medicine, Department of Cardiovascular and Renal Research, Faculty of Health Sciences, University of Southern Denmark, Odense, Denmark; c Department of Cancer and Inflammation, University of Southern Denmark, Odense, Denmark; d Department of Chemical Engineering, Faculty of Engineering, Imperial College London, London, UK; e Department of Metabolomics, Digestion and Reproduction, Faculty of Medicine, Imperial College London, London, UK

**Keywords:** Surfactant protein A, Surfactant protein D, Recombinant trimeric fragment, Collectin, Solubility tag, Respiratory distress syndrome, Surfactant, Therapeutics

## Abstract

Surfactant treatment for neonatal respiratory distress syndrome has dramatically improved survival of preterm infants. However, this has resulted in a markedly increased incidence of sequelae such as neonatal chronic inflammatory lung disease. The current surfactant preparations in clinical use lack the natural lung defence proteins surfactant proteins (SP)-A and D. These are known to have anti-inflammatory and anti-infective properties essential for maintaining healthy non-inflamed lungs.

Supplementation of currently available animal derived surfactant therapeutics with these anti-inflammatory proteins in the first few days of life could prevent the development of inflammatory lung disease in premature babies. However, current systems for production of recombinant versions of SP-A and SP-D require a complex solubilisation and refolding protocol limiting expression at scale for drug development.

Using a novel solubility tag, we describe the expression and purification of recombinant fragments of human (rfh) SP-A and SP-D using *Escherichia coli* without the need for refolding. We obtained a mean (± SD) of 23.3 (± 5.4) mg and 86 mg (± 3.5) per litre yield of rfhSP-A and rfhSP-D, respectively. rfhSP-D was trimeric and 68% bound to a ManNAc-affinity column, giving a final yield of 57.5 mg/litre of highly pure protein, substantially higher than the 3.3 mg/litre obtained through the standard refolding protocol. Further optimisation of this novel lab based method could potentially make rfhSP-A and rfhSP-D production more commercially feasible to enable development of novel therapeutics for the treatment of lung infection and inflammation.

## 1 Introduction

Treatment of neonatal respiratory distress syndrome (RDS) with exogenous surfactant has dramatically increased survival of preterm infants (Horbar et al., 1993; Schoendorf and Kiely, 1997; Malloy and Freeman, 2000). However, the corollary to this is an increase in survivors after preterm birth living with ventilator related lung damage and oxygen toxicity. As a result, between 50–70% of extremely preterm neonates may go on to develop inflammatory and emphysematous-like lung damage. This can result in neonatal chronic lung disease and a prolonged requirement for oxygen throughout infancy and early childhood.

Natural lung surfactant is composed of a variety of phospholipids as well as surfactant proteins A (SP-A), SP-B, SP-C and SP-D (Perez-Gil, 2008). The main function of lipid surfactant and SP-B and SP-C is to lower the surface tension of the alveolar air-liquid interface at end expiration to prevent alveolar collapse and facilitate breathing. Contrastingly, SP-A and SP-D act mainly as essential innate immune defence proteins which have key roles in keeping the lung in a non-infected, hypo-responsive and non-inflamed state (Wright, 2005; Fakih et al., 2015).

There is a wealth of literature demonstrating that SP-A and SP-D are implicated in the innate clearance of viruses, fungi and gram negative and gram positive bacteria (Pastva et al., 2007; Watson et al., 2019; Ujma et al., 2019). Moreover, SP-A and SP-D have been shown to be involved in the clearance of dead and dying apoptotic cells and to have key immunomodulatory effects on dendritic cells, macrophages and T-cells (Borron et al., 1996; Lin et al., 2010). These functions are essential in maintaining the lung in a hyporesponsive state to prevent inflammatory damage of the thin alveolar-capillary membrane and minimise the recruitment of inflammatory cells which could compromise respiratory gas exchange (Bridges et al., 2000; Watson et al., 2020).

Natural-derived surfactant preparations available commercially currently are manufactured by organic solvent extracts from animal lungs and thus they do not contain the water-soluble SP-A and SP-D (Sato and Ikegami, 2012; Baroutis et al., 2003). Similarly, recombinant versions of SP-A and SP-D are not included in the new synthetic surfactant therapeutics (Sato and Ikegami, 2012; Salgado et al., 2014).

SP-A and SP-D are collectins, composed of an N-terminal region, a collagen-like tail, a neck and a globular ligand-binding head domain, also known as the carbohydrate recognition domain (CRD). These proteins form functional trimeric units which bind to carbohydrates in a calcium-dependent manner, alongside protein receptors (Hoppe and Reid, 1994; Zhang et al., 2001; Jakel et al., 2013). SP-A and SP-D trimers can further oligomerise into octadecamers or oligomers containing up to 32 trimeric units, respectively (Strang et al., 1986). Due to their large size and complex quaternary structure, the production of full-length recombinant SP-A and SP-D for therapeutic purposes has been problematic. This is due to obstacles such as low expression yields, requirement of eukaryotic expression systems, difficulties in handling and obtaining a defined oligomeric state, as well as a requirement for administration in EDTA to prevent agglomeration and preserve solubility (Salgado et al., 2014; Haagsman et al., 1990; Brown-Augsburger et al., 1996; Sato et al., 2010).

Smaller recombinant fragments of human (rfh) SP-A1 and SP-D which lack the majority of the collagen-like domain have been produced and shown to form functional trimeric units. These fragments consist of the carbohydrate recognition domain (CRD), the α-helical neck domain and a short segment of eight G-X-Y repeats from the collagen domain. rfhSP-D has been well characterized structurally and functionally and demonstrated to maintain many of the functions of the full length protein (Watson et al., 2017; Clark, 2010; Madan et al., 2001). rfhSP-A has been produced and shown to be effective at neutralizing respiratory syncytial virus *in vitro* (Watson et al., 2017). These fragments have therapeutic potential due to their known consistent trimeric structure, their capacity to be stored and administered as a trimer in saline and their ability to be produced in cheaper and potentially more scalable prokaryotic expression systems (Clark, 2010; Bill, 2014).

rfhSP-A and rfhSP-D are currently expressed as insoluble proteins which require a solubilisation and refolding process (Watson et al., 2017; Littlejohn et al., 2018). This is a time-consuming process which is difficult to scale and leads to the majority of the protein being lost due to precipitation (Kaur et al., 2018). To overcome this problem, we looked to the N-terminal domain (NT) from a spider silk protein which is emerging as Nature’s own solubility tag to allow high expression levels of insoluble protein in a soluble form (Kronqvist et al., 2014; Hedhammar et al., 2008; Rising and Johansson, 2015; Kronqvist et al., 2017). The wildtype NT domain (NT_wt_) was previously shown to be an effective expression partner of rfhSP-A (Watson et al., 2017). This allowed high levels of expression, albeit of insoluble protein. Here we used a modified NT mutant (NT*) with increased solubility compared to NT_wt_. We used this to express high yields of rfhSP-A and rfhSP-D as soluble proteins under native conditions. This may facilitate the development of natural or synthetic surfactant preparations with the physiological composition of natural surfactant by supplementation with functional fragments of SP-A and SP-D for treatment of neonatal respiratory distress syndrome and other inflammatory lung diseases.

## 2 Materials and methods

### 2.1 Cloning

**Fig. 1:**
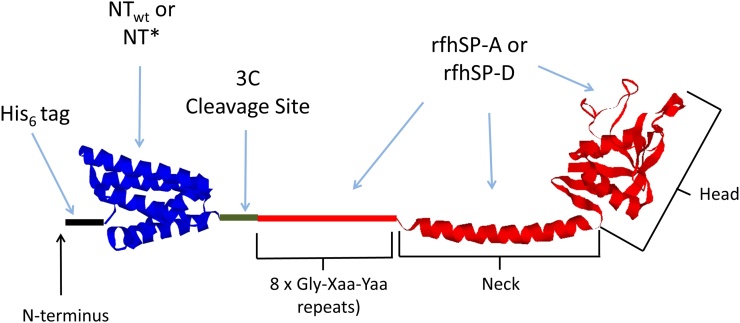
A schematic representing rfhSP-A and rfhSP-D fusion proteins with solubility tags NT_wt_ and NT*. From N-terminus to C-terminus the fusion proteins include: a His6-tag for purification of fusion proteins and removal of NT_wt_ or NT* once cleaved (black), NT_wt_ or NT* solubility tag (blue), a 3C protease cleavage site for removal of NT_wt_ and NT* (green) and rfhSP-A or rfhSP-D (red). rfhSP-A and rfhSP-D included 8 x Gly Xaa Yaa repeats, the neck and ligand binding head. The crystal structure for rfhSP-D was chosen for this figure to represent both rfhSP-A and rfhSP-D as rfhSP-A has not yet been crystallised (Littlejohn et al., 2018). This is an illustrative cartoon and the orientation, structure and scale may not be accurate (For interpretation of the references to colour in this figure legend, the reader is referred to the web version of this article).

The rfhSP-A1 gene was amplified using PCR. This was subsequently sub-cloned into a pT7 expression vector as a fusion gene with His_6_-NT_wt_ or His_6_-NT*, with a 3C protease cleavage site between the two genes on the N-terminal side of rfhSP-A or rfhSP-D (Fig. 1). Standard cleavage and ligation procedures were then used to make the fusion gene constructs (Watson et al., 2017). Constructs were then used to transform chemically competent *E. coli* BL21 (DE3).

### 2.2 Protein expression

Glycerol stocks of transformed *E. coli* BL21 (DE3) were grown in LB medium containing 70 mg/L kanamycin. Initial growth was overnight at 37 °C with shaking (180 rpm). 5 mL of this culture was then used to inoculate a fresh 500 mL of LB medium, containing 70 mg/L kanamycin which was grown at 30 °C with shaking (180 rpm) until the OD_600_ was ∼1. Expression was induced by addition of isopropyl-β-d-1-thiogalactopyranoside (IPTG) to a concentration of 0.5 mM, expression was undertaken overnight at 20 °C with shaking (180 rpm). After harvesting the cells through centrifugation at 4,000 x g for 20 min, the pellet was resuspended in 30 mL of 20 mM Tris−HCl, pH 8.0 and the cell solution was stored at -20 °C for at least 24 h.

### 2.3 Purification of rfhSP-A and rfhSP-D

Cell suspensions were defrosted and inclusion bodies were harvested through centrifugation at 7,200 x g for 40 min (4 °C). The pellet was subsequently suspended in 20 mM Tris−HCl, 1 mM CaCl_2_, 2 M urea, pH 8.0 and sonicated for 1 s pulses at 80% amplitude (for a total of 2 min) at 4 °C. Insoluble fractions were then removed through centrifugation at 30,000 x g for 30 min. To purify the protein, the supernatant was applied to a Ni-sepharose column (GE Healthcare) which had been equilibrated in 20 mM Tris−HCl, 1 mM CaCl_2_, 2 M Urea, pH 8.0. Washing of the column was undertaken using 20 mM Tris−HCl, 5 mM imidazole at 4 °C with a decreasing amount of urea (2 M, 1 M, 0.5 M and no urea). The bound fusion protein was then eluted using 20 mM Tris−HCl with 300 mM imidazole, pH 8.0. Imidazole was removed through dialysis at 4 °C overnight using 20 mM Tris−HCl, pH 8. After concentration of the protein, it was cleaved in 20 mM Tris−HCl, pH 8 with 1 mM DTT using 3C protease at a 1:10 ratio (w/w) for 6 h, at 4 °C. rfhSP-A and rfhSP-D were then purified through reapplying to the NI-sepharose to remove the His-tagged NT protein. rfhSP-D was purified as above but without the presence of urea.

### 2.4 Size-exclusion chromatography

Size exclusion chromatography was undertaken as previously described (Sorensen et al., 2009). Briefly, 200 μL of purified rfhSP-A or rfhSP-D was loaded onto a Superdex 200 h 10/30 column equilibrated in TBS with 5 mM EDTA, pH 7.4. The samples were run through at 0.3 mL/min and protein elution was detected through measuring optical absorbance at = 280 nm, this was compared to molecular weight standards including 12.4 kDa cytochrome c, 29 kDa carbonic anhydrase, 66 kDa BSA, 150 kDa alcohol dehydrogenase and 443 kDa apoferritin.

### 2.5 Affinity chromatography

ManNAc and mannan-sepharose columns (15 mL) were produced in Southampton, as previously described (Sorensen et al., 2009). rfhSP-A or rfhSP-D were applied to the column in 20 mM Tris, 150 mM NaCl (TBS) with 5 mM CaCl_2,_ pH 7.4 using an Äkta 900 system, as previously described (Watson et al., 2017). Columns were then washed in 20 mM Tris, 1 M NaCl, 5 mM CaCl_2,_ pH 7.4. Protein was eluted in TBS with 5 mM EDTA, pH 7.4.

### 2.6 Western blot

Western blot analysis was undertaken as previously described (Watson et al., 2017) using a monoclonal mouse α-native human (nh) SP-A primary HYB 238−04 (1 mg/mL), diluted 1:1,000 or a polyclonal rabbit α-rfhSP-D antibody (1.6 mg/mL), diluted 1:1,000 (Duvoix et al., 2011), to identify rfhSP-A and rfhSP-D, respectively. Detection was undertaken using a HRP-conjugated Goat α-Mouse IgG (H + L) antibody (Life Technologies, UK) (62–6520), diluted 1:10,000.

## 3 Results

### 3.1 Soluble expression of rfhSP-A and rfhSP-D using NT*

We previously used NT_wt_ as an expression tag to successfully over-express rfhSP-A in *E. coli* cells. However, this tag resulted in the protein residing within the inclusion body containing fraction, thus it required a subsequent solubilisation and refolding step using 8 M urea (Watson et al., 2017). Using removable fusion proteins NT_wt_ and NT* cloned to rfhSP-A and rfhSP-D (Fig. 1), we attempted to express these fragments as soluble proteins.

**Fig. 2:**
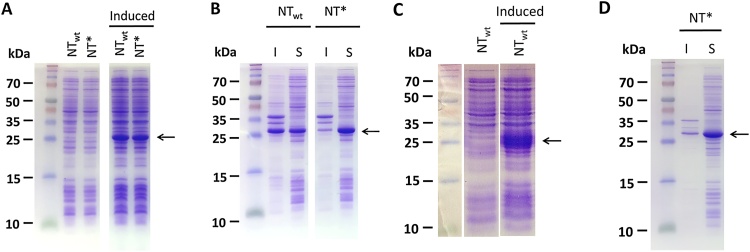
Expression of rfhSP-A and rfhSP-D as soluble proteins using NT_wt_ and NT*. Samples were analysed by SDS-PAGE, as compared to molecular standards. (A and C) Samples before and after IPTG induction (Induced) of (A) rfhSP-A or (C) rfhSP-D, both fused to either NT_wt_ or NT*. (B) After sonication and centrifugation, soluble (S) and insoluble (I) fractions containing NT_wt_-rfhSP-A (NT_wt_) and NT*-rfhSP-A (NT*) were analysed. (D) After sonication and centrifugation, soluble (S) and insoluble (I) fractions containing NT*-rfhSP-D (NT*) was analysed. (A, B and D) SDS-PAGE gels were run by Nina Kronqvist, Karolinska Institutet.

NT_wt_-rfhSP-A was expressed as a predominantly insoluble protein (Fig. 2A). However, through sonication using non-denaturing amounts of urea (2 M), almost 50% of the NT_wt_-rfhSP-A1 fusion protein could be obtained in the soluble fraction (Fig. 2B). NT* in fusion with rfhSP-A allowed for similarly high levels of protein expression, (Fig. 2A). However, NT* allowed for nearly all of the fusion protein to be expressed in the soluble fraction (Fig. 2B).

Both NT* and NT_wt_ allowed for high levels of rfhSP-D protein expression, the majority of which was expressed in the soluble fraction (Fig. 2C and D). Other solubility tags including Trx, OmpA and PelB were also used for comparison. However, these gave only low levels of expression of insoluble protein (data not shown).

### 3.2 Purification of rfhSP-A and rfhSP-D

**Fig. 3:**
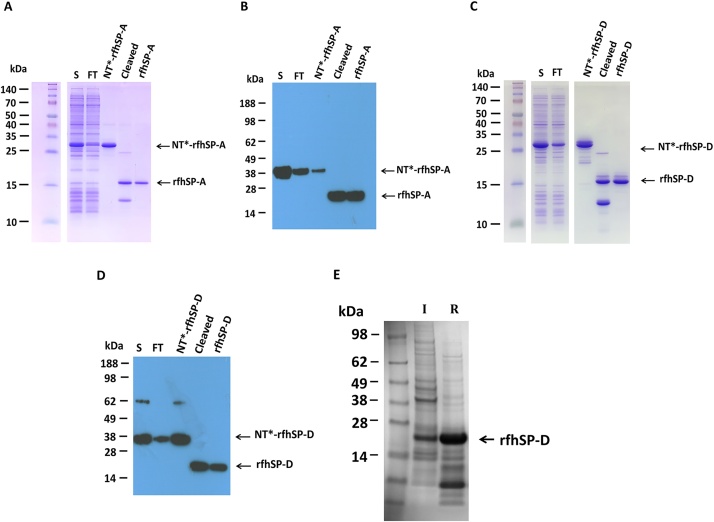
Purification of rfhSP-A and rfhSP-D using nickel affinity chromatography. (A–D) Protein samples taken during protein purification using nickel affinity column were analysed by SDS-PAGE. The soluble fraction (S) prior to application to the column and flow-through (FT) are indicated. Purified NT*-rfhSP-A and NT*-rfhSP-D eluted from the column are indicated (NT*-rfhSP-A and NT*-rfhSP-D). After cleavage of fusion proteins using 3C protease, the cleaved (cleaved) and purified rfhSP-A and rfhSP-D (rfhSP-A and rfhSP-D) were analysed by SDS-PAGE analysis. These samples were also analysed by western blot analysis using a (B) monoclonal mouse α-nhSP-A antibody to probe for rfhSP-A and (D) a polyclonal rabbit α-rfhSP-D antibody to probe for rfhSP-D. (E) rfhSP-D was also expressed, solubilised and refolded using the standard expression and purification protocol. Samples were taken after induction of whole bacterial cell lysis after rfhSP-D expression (I) and isolated protein after solubilisation and refolding (R). For all gels and westerns, molecular weights were compared with a protein standard. (A and C) SDS gels were run by Nina Kronqvist, Karolinska Institutet.

The NT*-rfhSP-A and NT*-rfhSP-D fusion constructs were cloned to contain an N-terminal His_6_ tag. This allowed purification of the fusion proteins by Nickel affinity purification. The purified NT*-rfhSP-A and NT*-rfhSP-D fusion proteins were subsequently cleaved with 3C protease to remove the NT* solubility tag (Fig. 1). After cleavage, rfhSP-A and rfhSP-D were then purified using a second round of nickel affinity purification. This removed the His_6_ tagged NT* and 3C enzyme using negative selection (Fig. 3).

Purification by nickel affinity purification resulted in a highly pure rfhSP-A, with no observable contamination from NT* or any other protein, as determined by SDS-PAGE (Fig. 3 A and C). rfhSP-A was confirmed using western blot analysis for detection of rfhSP-A using a monoclonal antibody against native human SP-A (Fig. 3 B). Upon cleavage of NT*-rfhSP-D, an additional higher order contaminating band was also seen (Fig. 3 C). However, this was not recognized by the rfhSP-D western blot and was removed with subsequent purification by affinity chromatography (Fig. 3D and 5C). The identity of rfhSP-A and rfhSP-D were confirmed using mass spectrometry (data not shown).

This streamline purification technique of solubly expressed protein yielded a mean (± SD) of 23.3 (± 5.4) mg (n = 4) of highly pure rfhSP-A and 86 mg (± 3.5) of rfhSP-D (n = 3) per litre of bacteria. This compared with the expression of rfhSP-D using the standard protocol which required time consuming solubilisation and refolding steps and yielded only 15.75 (± 1.06) (n = 2) of total protein, which was highly contaminated with bacterial proteins (Fig. 3E).

### 3.3 rfhSP-A and rfhSP-D produced using NT* are trimeric

**Fig. 4:**
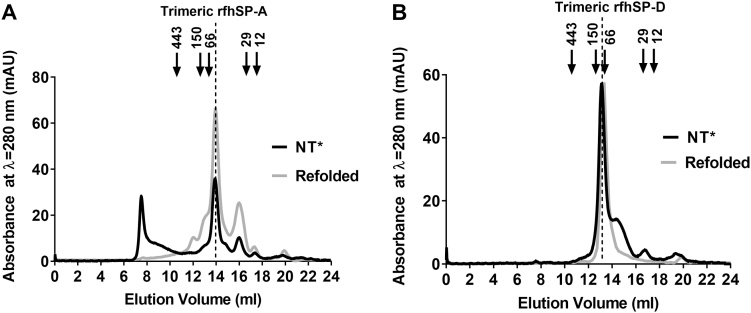
rfhSP-D produced using NT* is trimeric. The oligomeric structures of purified rfhSP-A and rfhSP-D after expression and purification using NT_wt_ and NT* were analysed by size-exclusion chromatography. Shown are the milli absorbance units at λ = 280 nm upon elution of protein from the column. Elution volumes were compared with various protein standards including 12.4 kDa cytochrome c, 29 kDa carbonic anhydrase, 66 kDa BSA, 150 kDa alcohol dehydrogenase and 443 kDa apoferritin. Indicated are the peaks corresponding to previously expressed rfhSP-A and purified rfhSP-D using the standard refolding protocol. (A) A chromatograph of rfhSP-A expressed and isolated as a soluble protein using (NT*) and rfhSP-A refolded using the previous refolding protocol using NT_wt_ (Refolded). (B) A chromatograph of rfhSP-D expressed and isolated as a soluble protein using (NT*) and rfhSP-D expressed and purified without an expression tag using the standard rfhSP-D purification protocol (Refolded).

The formation of trimeric units is essential for the biological activities of SP-A and SP-D. Thus to determine the trimeric structure of purified rfhSP-A and rfhSP-D produced using NT*, they were analysed using size-exclusion chromatography. Size-exclusion chromatography demonstrated that a mean (± SD) proportion of 24 (± 4.3)% (n = 4) of rfhSP-A produced using NT* eluted at the expected volume for trimeric rfhSP-A. This aligned with trimeric rfhSP-A produced using the previously used solubilisation and refolding protocol (Fig. 4A). However, a large proportion of rfhSP-A also had a higher apparent molecular weight of >443 kDa.

Comparatively 88.5 (± 4.0, n = 4)% of rfhSP-D expressed and purified using NT* eluted at the expected volume for trimeric rfhSP-D aligning with the elution volume of ManNAc purified rfhSP-D produced by our previous solubilisation and refolding protocol (Fig. 4B).

### 3.4 rfhSP-D produced using NT* is functional in binding to ManNAc

**Fig. 5:**
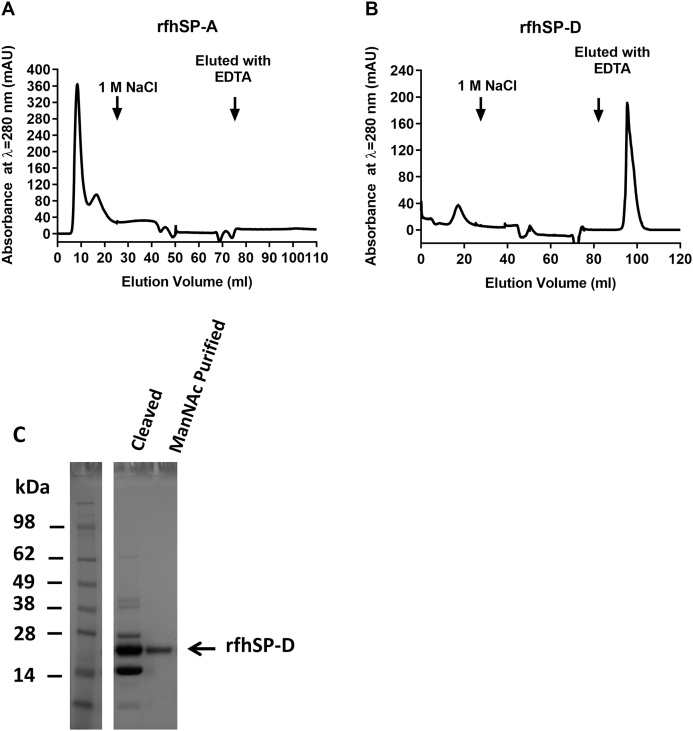
rfhSP-D produced using NT* is functional in binding to ManNAc. (A) rfhSP-A or (B) rfhSP-D expressed and purified using NT* were applied to a 15 mL ManNAc affinity column equilibrated in TBS in 5 mM CaCl_2_. After washing in 20 mM Tris, 1 M NaCl with 5 mM CaCl2, bound protein was eluted specifically using TBS with 5 mM EDTA. Shown are the chromatographs illustrating the milli absorbance units at λ = 280 nm upon elution of protein from the column. (C) rfhSP-D eluted from the ManNAc affinity column (ManNAc purified) was isolated and analysed by SDS-PAGE. This was compared with samples taken immediately after cleavage of NT*-rfhSP-D into NT* and rfhSP-D using 3C protease (cleaved). Indicated is the band corresponding to functional rfhSP-D which was purified by ManNAc affinity chromatography.

To demonstrate the functionality of rfhSP-A and rfhSP-D in binding to carbohydrates in a calcium-dependent manner, they were further purified using ManNAc-affinity chromatography. rfhSP-A produced using NT* did not bind to ManNAc-coupled sepharose columns or other carbohydrate columns including mannan and maltose (Fig. 5A ). However, 68% of the rfhSP-D purified using NT* did bind to a ManNAc column in a calcium-dependent manner; this was able to be eluted specifically in the presence of EDTA (Fig. 5B).

Elution of rfhSP-D from the ManNAc column yielded a highly pure rfhSP-D preparation, as determined by SDS-PAGE (Fig. 5C). Manufacture using NT* with subsequent ManNAc affinity chromatography resulted in 57.5 mg/Litre of functional trimeric rfhSP-D. This is substantially higher than the 3.3 mg of ManNAc purified rfhSP-D produced using the standard solubilisation and refolding protocol.

## 4 Discussion

Functional trimeric SP-A and SP-D fragments could have therapeutic potential in limiting ventilator and oxygen induced lung inflammation in preterm infants to help reduce the development of neonatal chronic lung disease. However, the current expression and purification systems of rfhSP-A and rfhSP-D require a solubilisation and refolding process which is time-consuming and difficult to undertake on an industrial scale (Kaur et al., 2018). Here we demonstrate the over expression and purification of rfhSP-A and rfhSP-D in a streamline process using a novel solubility tag, NT*. Furthermore we demonstrate that rfhSP-D produced by NT* is trimeric and functional in binding to ManNAc in a calcium-dependent manner.

We have demonstrated a novel expression system for the streamline production of pure, trimeric and functional rfhSP-D which gave a final yield of 57.5 mg/litre after ManNAc affinity purification. This is substantially higher than the 3.3 mg/litre obtained using the standard rfhSP-D process where 80–90% of solubilised protein is lost during refolding due to precipitation (Knudsen et al., 2007). This system also allowed the over expression and purification of rfhSP-A as a soluble protein, giving a yield of 23.3 (± 5.4) mg/litre, higher than the ∼12 mg/litre of yields than previously obtained during expression with NT_wt_ and refolding (Watson et al., 2017).

The majority of rfhSP-D expressed using this novel solubility tag was trimeric and functional in binding to ManNAc. However, only a quarter of rfhSP-A was of trimeric structure and no rfhSP-A bound to ManNAc, mannan or maltose affinity columns. This contrasts to rfhSP-A previously expressed as an insoluble protein using NT_wt_ where a fraction of refolded rfhSP-A did bind to carbohydrate affinity columns. This difference in functionality could be due to the different expression environment in the soluble fraction of the bacterial cell compared to that during the refolding step (Heath et al., 2015; Schlegel et al., 2013). Furthermore, variation of expression and purification parameters could have an impact on the functionality of the end product and this remains to be understood (Schlegel et al., 2013). As compared with rfhSP-D, rfhSP-A required sonication in 2 M urea to allow dissociation from the inclusion bodies. Although this is unlikely to denature the protein, this could lead to slight alterations in the rfhSP-A structure which could impact on functionality. Requirement for 2 M urea is likely required due to the rfhSP-A interacting with the insoluble *E. coli* cell fraction. SP-A is known to be inherently more lipophilic than SP-D, hence SP-A is frequently purified by butanol extraction (Watson et al., 2017). Purification of rfhSP-A expressed as a soluble protein using 2 M is different to the previously used refolding process in the presence of glycerol, which may stabilise the CRD. Further development purifying rfhSP-A in different solutions with various additives could be tested to try to obtain functional rfhSP-A using this novel approach. Furthermore, modification of expression conditions as well as the bacterial strains used could be trialled to optimise the soluble protein expression.

Notably, although this allows the soluble expression of higher yields of rfhSP-A and rfhSP-D than previously possible, this is still the first iteration of a lab scale system. Preliminary work expressing NT*-rfhSP-D with a different enzyme cleavage site allowed for a >70% higher initial yield (Watson, 2016). However this construct could not be used due to non-specific enzymatic cleavage. This alternative construct which results in higher expression yields demonstrates that there is likely scope to further optimise constructs and expression conditions to obtain improved yields. Through further optimization, process development and use of industrial fermenters, it is likely that a yield of rfhSP-D in the range of grams per litre could be obtained, as has been done for other heterologous proteins (Fordjour et al., 2019; Lu et al., 2015). There are clear advantages of expressing rfhSP-A and rfhSP-D in *E. coli* due to cost and yield (Bill, 2014; Kaur et al., 2018). Other expression systems such as the yeast expression system using Pichia pastoris have previously been used to express rfhSP-D relatively cheaply and in high yields and may merit further investigation as to scalability (Hakansson et al., 1999). In this present soluble bacterial expression system, NT* is cleaved from rfhSP-D through addition of an enzyme. This is removed through facilitated purification and incorporation of a His_6_ tag. However, the impact of adding multiple steps on the scalability of this laboratory based process also merits consideration.

## 5 Conclusions

We have used a novel soluble expression system to allow over-expression of high levels of soluble rfhSP-A and rfhSP-D. This advance increases the feasibility of further detailed investigations on the structure function relationships of recombinant fragments of SP-A and SP-D compared to the native proteins. Furthermore, it represents a significant step forward to scalable development of rfhSP-D and rfhSP-A as novel therapeutics for the treatment of lung infection and inflammation.

## Funding

This work was supported by the Medical Research Council (MRC), UK.

## CRediT authorship contribution statement

**Alastair Watson:** Conceptualization, Methodology, Data curation, Formal analysis, Investigation, Project administration, Writing - original draft. **Grith L. Sørensen:** Investigation. **Uffe Holmskov:** Investigation. **Harry J. Whitwell:** Formal analysis, Investigation. **Jens Madsen:** Funding acquisition, Supervision, Validation, Conceptualization, Methodology, Writing - review & editing. **Howard Clark:** Funding acquisition, Supervision, Validation, Conceptualization, Methodology, Writing - review & editing.

## Declaration of Competing Interest

A patent has been jointly filed by University of Southampton and Spiber Technologies (WO2017109477A2·2017−06-29) on which Alastair Watson, Jens Madsen and Howard Clark are named inventors. The NT technology was developed and is owned by Spiber Technologies.

## References

[bib0005] Baroutis G., Kaleyias J., Liarou T., Papathoma E., Hatzistamatiou Z., Costalos C. (2003). Comparison of three treatment regimens of natural surfactant preparations in neonatal respiratory distress syndrome. Eur. J. Pediatr..

[bib0010] Bill R.M. (2014). Playing catch-up with Escherichia coli: using yeast to increase success rates in recombinant protein production experiments. Front. Microbiol..

[bib0015] Borron P., Veldhuizen R.A., Lewis J.F., Possmayer F., Caveney A., Inchley K. (1996). Surfactant associated protein-A inhibits human lymphocyte proliferation and IL-2 production. Am. J. Respir. Cell Mol. Biol..

[bib0020] Bridges J.P., Davis H.W., Damodarasamy M., Kuroki Y., Howles G., Hui D.Y. (2000). Pulmonary surfactant proteins A and D are potent endogenous inhibitors of lipid peroxidation and oxidative cellular injury. J. Biol. Chem..

[bib0025] Brown-Augsburger P., Chang D., Rust K., Crouch E.C. (1996). Biosynthesis of surfactant protein D. Contributions of conserved NH2-terminal cysteine residues and collagen helix formation to assembly and secretion. J. Biol. Chem..

[bib0030] Clark H.W. (2010). Untapped therapeutic potential of surfactant proteins: is there a case for recombinant SP-D supplementation in neonatal lung disease?. Neonatology..

[bib0035] Duvoix A., Mackay R.M., Henderson N., McGreal E., Postle A., Reid K. (2011). Physiological concentration of calcium inhibits elastase-induced cleavage of a functional recombinant fragment of surfactant protein D. Immunobiology..

[bib0040] Fakih D., Pilecki B., Schlosser A., Jepsen C.S., Thomsen L.K., Ormhoj M. (2015). Protective effects of surfactant protein D treatment in 1,3-beta-glucan-modulated allergic inflammation. Am. J. Physiol. Lung Cell Mol. Physiol..

[bib0045] Fordjour E., Adipah F.K., Zhou S., Du G., Zhou J. (2019). Metabolic engineering of Escherichia coli BL21 (DE3) for de novo production of L-DOPA from D-glucose. Microb. Cell Fact..

[bib0050] Haagsman H.P., Sargeant T., Hauschka P.V., Benson B.J., Hawgood S. (1990). Binding of calcium to SP-A, a surfactant-associated protein. Biochemistry..

[bib0055] Hakansson K., Lim N.K., Hoppe H.J., Reid K.B. (1999). Crystal structure of the trimeric alpha-helical coiled-coil and the three lectin domains of human lung surfactant protein D. Structure.

[bib0060] Hedhammar M., Rising A., Grip S., Martinez A.S., Nordling K., Casals C. (2008). Structural properties of recombinant nonrepetitive and repetitive parts of major ampullate spidroin 1 from Euprosthenops australis: implications for fiber formation. Biochemistry..

[bib0065] Hoppe H.J., Reid K.B.M. (1994). Collectins - soluble proteins containing collagenous regions and lectin domains - and their roles in innate immunity. Protein Sci..

[bib0070] Horbar J.D., Wright E.C., Onstad L. (1993). Decreasing mortality associated with the introduction of surfactant therapy: an observational study of neonates weighing 601 to 1300 grams at birth. The Members of the National Institute of Child Health and Human Development Neonatal Research Network. Pediatrics.

[bib0075] Jakel A., Qaseem A.S., Kishore U., Sim R.B. (2013). Ligands and receptors of lung surfactant proteins SP-A and SP-D. Front. Biosci. Landmark Ed. (Landmark Ed).

[bib0080] Kaur J., Kumar A., Kaur J. (2018). Strategies for optimization of heterologous protein expression in E. coli: roadblocks and reinforcements. Int. J. Biol. Macromol..

[bib0085] Knudsen L., Ochs M., Mackay R., Townsend P., Deb R., Muhlfeld C. (2007). Truncated recombinant human SP-D attenuates emphysema and type II cell changes in SP-D deficient mice. Respir. Res..

[bib0090] Kronqvist N., Otikovs M., Chmyrov V., Chen G., Andersson M., Nordling K. (2014). Sequential pH-driven dimerization and stabilization of the N-terminal domain enables rapid spider silk formation. Nat. Commun..

[bib0095] Kronqvist N., Sarr M., Lindqvist A., Nordling K., Otikovs M., Venturi L. (2017). Efficient protein production inspired by how spiders make silk. Nat. Commun..

[bib0100] Heath C.J., del Mar Cendra M., Watson A. (2015). Co-transcriptomes of initial interactions in vitro between streptococcus pneumoniae and human pleural mesothelial cells. PloS One.

[bib0105] Lin K.W., Jen K.Y., Suarez C.J., Crouch E.C., Perkins D.L., Finn P.W. (2010). Surfactant protein D-mediated decrease of allergen-induced inflammation is dependent upon CTLA4. Journal of immunology (Baltimore, Md: 1950).

[bib0110] Littlejohn J.R., da Silva R.F., Neale W.A., Smallcombe C.C., Clark H.W., Mackay R.A. (2018). Structural definition of hSP-D recognition of Salmonella enterica LPS inner core oligosaccharides reveals alternative binding modes for the same LPS. PLoS One.

[bib0115] Lu J., Song Q., Ji Z., Liu X., Wang T., Kang Q. (2015). Fermentation optimization of maltose-binding protein fused to neutrophil-activating protein from Escherichia coli TB1. Electron. J. Biotechnol..

[bib0120] Madan T., Kishore U., Singh M., Strong P., Hussain E.M., Reid K.B. (2001). Protective role of lung surfactant protein D in a murine model of invasive pulmonary aspergillosis. Infect. Immun..

[bib0125] Malloy M.H., Freeman D.H. (2000). Respiratory distress syndrome mortality in the United States, 1987 to 1995. J. Perinatol..

[bib0130] Pastva A.M., Wright J.R., Williams K.L. (2007). Immunomodulatory roles of surfactant proteins A and D: implications in lung disease. Proc. Am. Thorac. Soc..

[bib0135] Perez-Gil J. (2008). Structure of pulmonary surfactant membranes and films: the role of proteins and lipid-protein interactions. Biochim. Biophys. Acta.

[bib0140] Rising A., Johansson J. (2015). Toward spinning artificial spider silk. Nat. Chem. Biol..

[bib0145] Salgado D., Fischer R., Schillberg S., Twyman R.M., Rasche S. (2014). Comparative evaluation of heterologous production systems for recombinant pulmonary surfactant protein D. Front. Immunol..

[bib0150] Sato A., Ikegami M. (2012). SP-B and SP-C containing new synthetic surfactant for treatment of extremely immature lamb lung. PLoS One.

[bib0155] Sato A., Whitsett J.A., Scheule R.K., Ikegami M. (2010). Surfactant protein-d inhibits lung inflammation caused by ventilation in premature newborn lambs. Am. J. Respir. Crit. Care Med..

[bib0160] Schlegel S., Rujas E., Ytterberg A.J., Zubarev R.A., Luirink J., de Gier J.W. (2013). Optimizing heterologous protein production in the periplasm of E. Coli by regulating gene expression levels. Microb. Cell Fact..

[bib0165] Schoendorf K.C., Kiely J.L. (1997). Birth weight and age-specific analysis of the 1990 US infant mortality drop. Was it surfactant?. Arch. Pediatr. Adolesc. Med..

[bib0170] Sorensen G.L., Hoegh S.V., Leth-Larsen R., Thomsen T.H., Floridon C., Smith K. (2009). Multimeric and trimeric subunit SP-D are interconvertible structures with distinct ligand interaction. Mol. Immunol..

[bib0175] Strang C.J., Slayter H.S., Lachmann P.J., Davis A.E. (1986). Ultrastructure and composition of bovine conglutinin. Biochem. J..

[bib0180] Ujma S., Carse S., Chetty A., Horsnell W., Clark H., Madsen J. (2019). Surfactant protein a impairs genital HPV16 pseudovirus infection by innate immune cell activation in a murine model. Pathogens..

[bib0185] Watson A. (2016). Recombinant Expression of Functional Trimeric Fragments of Human SP-A and SP-D.

[bib0190] Watson A., Kronqvist N., Spalluto C.M., Griffiths M., Staples K.J., Wilkinson T. (2017). Novel expression of a functional trimeric fragment of human SP-A with efficacy in neutralisation of RSV. Immunobiology..

[bib0195] Watson A., Phipps M.J., Clark H.W., Skylaris C.-K., Madsen J. (2019). Surfactant proteins a and d: trimerized innate immunity proteins with an affinity for viral fusion proteins. J. Innate Immun..

[bib0200] Watson A., Spalluto C.M., McCrae C. (2020). Dynamics of IFN-β responses during respiratory viral infection: insights for therapeutic strategies. Am. J. Respir. Crit. Care Med..

[bib0205] Wright J.R. (2005). Immunoregulatory functions of surfactant proteins. Nat. Rev. Immunol..

[bib0210] Zhang P.N., McAlinden A., Li S., Schumacher T., Wang H.L., Hu S.S. (2001). The amino-terminal heptad repeats of the coiled-coil neck domain of pulmonary surfactant protein D are necessary for the assembly of trimeric subunits and dodecamers. J. Biol. Chem..

